# Whole-exome sequencing of a pedigree segregating asthma

**DOI:** 10.1186/1471-2350-13-95

**Published:** 2012-10-09

**Authors:** Andrew T DeWan, Kathryn Brigham Egan, Karen Hellenbrand, Keli Sorrentino, Nicole Pizzoferrato, Kyle M Walsh, Michael B Bracken

**Affiliations:** 1 Department of Chronic Disease Epidemiology, Yale School of Public Health, 60 College St, New Haven, CT 06520, USA; 2 Center for Perinatal, Pediatric and Environmental Epidemiology, Yale University Schools of Public Health and Medicine, 1 Church Street, New Haven, CT 06510, USA; 3 Current affiliation: Division of Cancer Epidemiology, University of California, San Francisco, CA, USA

**Keywords:** Asthma, Whole-exome sequencing, PDE4DIP, CBLB, KALRN

## Abstract

**Background:**

Despite the success of genome-wide association studies for asthma, few, if any, definitively causal variants have been identified and there is still a substantial portion of the heritability of the disease yet to be discovered. Some of this “missing heritability” may be accounted for by family-specific coding variants found to be segregating with asthma.

**Methods:**

To identify family-specific variants segregating with asthma, we recruited one family from a previous study of asthma as reporting multiple asthmatic and non-asthmatic children. We performed whole-exome sequencing on all four children and both parents and identified coding variants segregating with asthma that were not found in other variant databases.

**Results:**

Ten novel variants were identified that were found in the two affected offspring and affected mother, but absent in the unaffected father and two unaffected offspring. Of these ten, variants in three genes (*PDE4DIP*, *CBLB*, and *KALRN*) were deemed of particular interest based on their functional prediction scores and previously reported function or asthma association. We did not identify any common risk variants segregating with asthma, however, we did observe an increase in the number of novel, nonsynonymous variants in asthma candidate genes in the asthmatic children compared to the non-asthmatic children.

**Conclusions:**

This is the first report applying exome sequencing to identify asthma susceptibility variants. Despite having sequenced only one family segregating asthma, we have identified several potentially functional variants in interesting asthma candidate genes. This will provide the basis for future work in which more families will be sequenced to identify variants across families that cluster within genes.

## Background

The genome-wide association approach to uncovering the genetic factors influencing common phenotypes is premised on the “common disease, common variant” hypothesis in which alleles that are common in the population (minor allele frequency [MAF] > 1%) will be associated with these phenotypes. At least twelve genome-wide association studies (GWAS) of asthma have been conducted and have yielded numerous associations, with the most significant (and in most cases replicated) associations occurring in or near the following genes: *ORMDL3*[1,2], *PDE4D*[3], *HLA-DRB1*[2], *HLA-DQ*[2,4], *RAD50-IL13*[4], *DENND1B*[5], *TLE4*[6], *SMAD3*[2], *IL1RL1*[7], *IL18R1*[2], *IL33*[2], *IL2RB*[2], *RORA*[2], and *SLC22A5*[2]. These findings have greatly expanded our understanding of the disease, having identified several novel genetic loci that had never previously been implicated in the pathogenesis of asthma (e.g. *ORMDL3*, *RAD50*, *DENND1B*, *TLE4*). Despite these successes, no definitive causal variants have been identified in any of these genes. It is asserted that the associated variants are in LD with the causal variants in these genes, but more effort must be made to identify causal variants so that we can begin to understand the biology of these genes in the etiology of asthma.

It is now recognized that GWAS are not able to identify all or even the majority of the genetic variants contributing to a disease phenotype [8-10]. The proportion of the heritability of a phenotype not explained by known genetic variants has been termed the “missing heritability.” AMD is so far the disease with the highest proportion of heritability explained by known loci, but even then only explains ~50% of the heritability [11]. Large sample sizes have allowed us to identify loci with small effect sizes yielding p-values well below the typical 5 × 10^-8^, required for a scan of one million single-nucleotide polymorphisms (SNPs), but we are no longer identifying major contributors to phenotype variation. In the case of height, across two studies, a total of 60,000 subjects were genotyped, but significantly associated, and replicated, variants in two different genes only explained between 0.3 and 0.5% of the variation [12,13]. For asthma, the population attributable fraction estimates for known asthma loci range from 3.9 to 24% [14], while explaining more risk than typical height loci, still indicate a substantial proportion of missing heritability. This suggests that a paradigm shift in our search for susceptibility loci for common diseases is warranted.

One explanation for the lack of functional variant identification is that the degree of genetic heterogeneity for common diseases is markedly higher than previously thought, possibly due to the presence of rare or even family-specific mutations with a large effect [15]. Our hypothesis is that individual families segregate “family-specific” variants contributing to asthma susceptibility and that at least one family-specific variant is necessary but not sufficient for disease development within individuals in the family. It is when these family-specific mutations occur in the context of common asthma susceptibility variants that the disease will develop.

To identify family-specific variants segregating with asthma, we recruited one family originally identified via one asthmatic child enrolled in the Perinatal Risk of Asthma in Infants of Asthmatic Mothers (PRAM) study [16] as reporting multiple asthmatic and non-asthmatic children. We performed whole-exome sequencing on all four children and both parents.

## Methods

### Patient recruitment/sample collection

The recruitment, questionnaire, blood collection and exome sequencing were approved by the Yale University Human Investigation Committee. The family was identified from information collected as part of the Perinatal Risk of Asthma in Infants of Asthmatic Mothers (PRAM), a study designed to assess the risk of asthma in children born to asthmatic mothers. We identified one family in this study that originally reported having two asthmatic children and two non-asthmatic children. The family was recontacted by a research associate to explain the study and determine the family’s interest in participating. A trained interviewer and phlebotomist visited the family in their home to conduct the interview and collect blood samples from each family member. The phlebotomist drew 10mL of blood from each family member in purple top EDTA tubes. The phlebotomist stored the blood on ice and delivered it to the lab within 24 hours. Each blood sample was divided into 1mL aliquots. DNA was extracted from one 1mL aliquot using the QIAGEN Blood Maxi kit. Each DNA sample was run on a 1% agarose gel to determine if it was of high quality (strong high molecular weight [> 10kb] band and no smear). DNA was quantified using TaqMan RNase P Detection Reagent Kit (Applied Biosystems).

### Phenotype collection

The interviewer conducted an interview with each family member to assess their asthma diagnosis, symptoms and medication. For each subject, the interviewer asked questions about their asthma and/or allergies. The subject was asked about asthma diagnosis by a physician, date of diagnosis and name of physician. Whether or not they had been diagnosed, the subject was asked about any of the following symptoms: wheeze, persistent cough, chest tightness or shortness of breath. A detailed description of the frequency and duration of symptoms for the past 12 months was obtained. If the subject had not experienced symptoms in the past 12 months, then the dates when symptoms last occurred, and a description of their frequency and duration at that time was recorded. The interviewer recorded all asthma medications used in the past 12 months; route of administration (nebulizer, inhaler, oral) and frequency of use. If no medication had been used in the past year, the date medication was last used, name of the medication and frequency of use were ascertained. Questions were asked about the occurrence and frequency of upper respiratory infections in the past 12 months, and life-time lower respiratory infections, particularly: respiratory syncytial virus (RSV), bronchitis, bronchiolitis, pneumonia and croup. Information was gathered about hospitalizations and emergency room visits for asthma, and other respiratory illnesses. Physician visits in the past 12 months and treatment by a pulmonologist, allergist or asthma specialist were recorded. Questions were asked to determine whether their physical activity is limited by asthma, or asthma symptoms. For children, the home interview also obtained information on post-natal risk factors for asthma development (e.g. newborn intensive care nursery stays).

### Sequencing

Five micrograms of DNA was submitted to the Yale Center for Genome Analysis. One microgram of genomic DNA was sheared to a mean fragment length of 140 base pairs using focused acoustic energy (Covaris E210, part #5000003). Fragmented DNA samples were then transferred to a 96-well plate and library construction was completed using a liquid handling robot (Caliper Sciclone, part #SG3-11020-0100). Magnetic AMPure XP beads (Beckman Coulter, part #63882) were used to purify the sheared DNA samples and remained with the sample throughout library construction. Following each process step, DNA was selectively precipitated by weight and re-bound to the beads through addition of a 20% polyethylene glycol, 2.5 M NaCl solution. Following fragmentation, T4 DNA polymerase and T4 polynucleotide kinase blunt ended and phosphorylated the fragments. The large Klenow fragment then added a single adenine residue to the 3' end of each fragment and custom adapters (IDT) were ligated using T4 DNA ligase. Adapter-ligated DNA fragments were then amplified via the polymerase chain reaction (PCR) using custom-made primers (IDT). During PCR, a unique 6 base index was inserted at one end of each DNA fragment. Sample concentration and insert size distribution were determined using the Caliper LabChip GX system (Caliper, part #122000/B). Samples yielding at least 1 μg of amplified DNA were used for capture.

Five hundred nanograms of prepared genomic DNA library was lyophilized with Cot-1 DNA and custom adapter blocking oligos (IDT). The dried sample was reconstituted according the manufacturer's protocol (Roche/Nimblegen), heat-denatured, and mixed with biotinylated DNA probes produced by Nimblegen (Nimblegen, SeqCap EZ Exome version 2, part #05860504001). Hybridizations were performed at 47°C for 68 hours. Once the capture was complete the samples were mixed with streptavidin-coated beads and washed with a series of stringent buffers to remove non-specifically bound DNA fragments. The captured fragments were PCR amplified and purified with AMPure XP beads. Capture efficiency was evaluated by quantitative PCR (Roche Light Cycler 480, part #5015243001). Equal amounts of pre- and post-capture libraries were evaluated at 4 sites to confirm successful exome enrichment and at 2 other sites to show non-exome de-enrichment in the captured sample relative to the pre-capture library. All samples met appropriate cut-offs for both and were quantified by qRT-PCR using a commercially available kit (KAPA Biosystems, part #KK4601). Insert size distribution was determined with the LabChip GX.

Sample concentrations were normalized to 2 nM, combined accordingly for the number of samples to be sequenced per lane, and loaded onto Illumina version 3 flow cells at a concentration that yields 170–200 million passing filter clusters. The samples were sequenced using 75bp paired-end sequencing on an Illumina HiSeq 2000 according to Illumina protocols. The 6 base pair index was read during an independent sequencing read that automatically follows the completion of read 1 and uses an additional sequencing primer (Illumina, part #15019606).

Signal intensities were converted to individual base calls on the machine during a run using the system's Real Time Analysis (RTA) software. Sample de-multiplexing was performed using Illumina's CASAVA 1.8 software suite and FASTQ files for each sample were produced.

### Alignment, variant identification and filtering

Reads were aligned using the Burrows-Wheeler Algorithm (BWA) [17] to the reference human genome (build 37). Alignments for each sample were converted to BAM format, sorted, indexed, PCR duplicates marked and then merged into one BAM file for all six sample using Samtools [18]. Alignments in the the combined BAM file were then locally realigned around insertions/deletions (indels), recalibrated, and variants called (SNPs and indels using the UnifiedGenotyper) for the all six samples together using the utilities in the Genome Analysis Toolkit (GATK) [19,20].

Variants were filtered and flagged as low quality using the following metrics: three or more variants detected within 10bp; four or more alignments map to different locations equally well; coverage less than five reads; quality score < 50; low quality for a particular sequence depth (variant confidence/unfiltered depth < 1.5); and strand bias (Phred-scaled p-values using Fisher’s Exact Test > 200). A variant flagged for any ONE of these filters was labeled ‘low quality’ and not considered further in this analysis.

### Annotation

Variants were annotated using ANNOVAR [21] for function (exonic or splicing); gene; exon function (synonymous, nonsynonymous, stopgain, nonframeshift or frameshift indel); amino acid change; conservation; allele frequency in 1000 Genomes Project; dbSNP reference number; functional prediction scores (SIFT [prediction of a change being damaging (> 0.95) or tolerated (< 0.95)], Polyphen2 [prediction if a change is damaging (> 0.85), possibly damaging (0.85-0.15) or benign (< 0.15)], LRT [likelihood ratio test for codon constraint ranging from 0–1 with larger scores indicating constrained], MutationTaster [prediction of a disease causing variant, 1-pvalue], PhyloP [prediction of a conserved (> 0.95) or non-conserved (< 0.95) site]) [22]; and chromosome position.

### Validation genotyping

To validate the genotypes of the variants in *PDE4DIP*, *CBLB* and *KALRN* we designed a custom TaqMan assay (Applied Biosystems) targeting each of these variants. Each sample was run in triplicate. Fourteen additional samples were run to aid in clustering genotypes and act as negative controls as they were only expected to be homozygous wild-type.

### Candidate gene variant counts

We previously identified 251 asthma candidate genes from the literature [23]. We queried the list of 38,103 variants that passed the QC filters to identify those variants that were annotated as being within or near one of these genes. For each subject, we counted the number of non-referent alleles at each variant annotated within one of the 251 genes. We then restricted this to only novel variants (not contained within dbSNP). Finally, we restricted this to novel and non-synonymous variants. In order to compute the statistical significance of the number of novel or novel and nonsynonymous variants among the case children compared to control, we used the Pearson *χ*2 test and also calculated odds ratios (OR) and 95% confidence intervals (95%CI). We compared the total number of novel rare alleles (n=16 among cases, n=8 among controls) to the total number of rare alleles identified minus the number of novel rare alleles (n=56767 among cases, n=55742 among controls). The same calculation was also performed for the variants classified as novel and nonsynonymous.

### *de novo* Variant quality control

Due to the difficulty identifying de novo variants from false positive genotype calls, we imposed strict quality control criteria. These were based, in part, on the quality control measures used in Neale et al. [24]. For a variant to be considered de novo we required it to meet the following criteria: 1) more than 10 reads for the mother, father and child carrying the *de novo* mutation; 2) mother or father could have no more than 5% of the total reads being from the non-referent allele; 3) if the *de novo* variant in the child was heterozygous, the variant could have no more than 70% of the reads being from the referent allele.

## Results

Two children reported a doctor’s diagnosis of asthma and allergies and one of the two also reported taking albuterol (up to 14 times per month) and a generic allergy medication (1–4 times per month) in the preceding 12 months. The other asthmatic child was reported (per the mother) as having wheeze and persistent cough during all seasons of the first year of life. The two remaining children reported not having a doctor’s diagnosis of asthma, no allergies and no asthma or allergy medication use. The mother reported having a doctor’s diagnosis of asthma and allergies, and taking albuterol daily or almost daily during the previous 12 months. The father reported not having a doctor’s diagnosis of asthma, but reported having allergies and taking a generic allergy medication 1–4 times in one of the preceding 12 months. See Table 1 for demographic and phenotypic details.

**Table 1:** Demographic and phenotype information

**Family member**	**Age**	**Asthma diagnosis**	**Allergies**	**Asthma duration (years)**	**Medications**
Mother	45	Yes	Yes	16	Albuterol
Father	46	No	Yes	NA	Generic Allergy
Child 1	11	Yes	Yes	Unknown^1^	None
Child 2	17	Yes	Yes	15	Albuterol, Generic Allergy
Child 3	15	No	No	NA	None
Child 4	9	No	No	NA	None

^1^Child and parents were unable to provide the age of diagnosis of asthma, but the mother reported the child being diagnosed with reactive airway disease between the ages of 1–2 years. The mother reported that this child had wheezing and persistent cough during the first year of life.

Overall, we attained high coverage of the exome, with four samples achieving >100 million 76 bp paired-end reads and the other two with more than 52 and 89 million reads (Table 2). For called variants, the average coverage was more than 100X for all subjects and among variants that passed all filters more than 110X (Table 2). It should be noted that Child 3 had fewer reads and lower coverage, however this only resulted in ~2% fewer called variants within exons that passed our stringent quality control filters. There were 55,370 variants called that were variable in one or more of the samples. After applying stringent quality control filters to eliminate low quality variants, 38,103 remained. The depth of coverage (*i.e.* the number of times an individual base was sequenced) of variants was high in all samples, with average per-sample depth of coverage of variants ranging from 92X to 210X among the high quality variants (Table 2). Of these high quality variants, the majority were within exons (66.3%), the 3’ untranslated regions (12.8%), introns (9%), 5’ untranslated regions (6%), or exons of non-coding RNAs (3.7%) (Table 3). Less than 1% of all high quality variants were in regions not considered to be within or around genes (Table 3).

**Table 2:** Number of reads, variants and variant coverage by sample

**Sample**	**Paired reads**^**2**^	**Unpaired reads**^**3**^	**Unmapped reads**^**4**^	**Read pair duplicates**^**5**^	**Unpaired read duplicates**^**6**^	**# Variants**^**7**^	**Avg. variant coverage**^**8**^	**#Variants: passed QC filters**^**9**^	**Avg. variant coverage: passed QC filters**^**10**^
Mother	89,967,478	2,061,484	10,764,768	8,577,327	1,636,019	36,275	156.0	28,232	148.7
Father	107,937,586	2,297,044	12,181,833	10,863,150	1,863,721	36,650	187.3	28,007	183.0
Child 1	105,231,048	2,088,137	10,883,542	12,455,453	1,705,727	37,267	174.7	28,370	169.6
Child 2	129,947,192	2,401,685	11,122,401	15,054,214	2,012,381	37,457	213.4	28,413	210.4
Child 3	52,005,299	932,213	3,544,678	2,870,062	634,587	34,826	100.4	27,376	92.9
Child 4	118,061,614	2,832,204	15,671,206	13,849,071	2,369,338	36,946	195.4	28,374	190.7
Total Unique^1^						55,370		38,103	

^1^Total number of unique variants across all six samples.

^2^Number of pairs of reads (reads from the opposite ends of the same DNA molecule) for which the corresponding read pair was identified.

^3^Number of reads for which the corresponding read pair was not identified.

^4^Number of reads that could not be mapped to the reference human genome.

^5^Number of read pairs that were the result of PCR duplicates.

^6^Number of unpaired reads that were the result of PCR duplicates.

^7^Total number of variants called by the Genome Analysis Toolkit (SNPs and indels).

^8^Average depth of coverage across all variants called for an individual sample.

^9^Number of variants that passed the quality control filters (see Alignment, Variant Identification and Filtering section above).

^10^Average depth of coverage across all variants that passed the quality control filters for an individual sample.

**Table 3:** Distribution of variant types

**Variant type**^**1**^	**All called variants**	**Variants passed QC filters**
EXONIC	32018	25278
UTR3	10106	4881
INTRONIC	4236	3428
UTR5	3387	2227
ncRNA_EXONIC	4164	1398
EXONIC_SPLICING	442	347
SPLICING	225	133
ncRNA_INTRONIC	179	90
ncRNA_UTR3	149	71
ncRNA_UTR5	39	21
UTR5_UTR3	8	7
ncRNA_SPLICING	31	6
UPSTREAM^2^	211	131
DOWNSTREAM^2^	113	60
INTERGENIC^2^	49	17
UPSTREAM_DOWNSTREAM^2^	13	8
Total	55,370	38,103

^1^Variant types as annotated by ANNOVAR [Abbreviations: UTR3 = 3’ Untranslated region; UTR5 = 5’ Untranslated region; ncRNA = non-coding RNA].

^2^Variants not considered to be within genes among all variants called (0.7%) and variant that passed the stringent QC filters (0.6%).

Given our interest in functional, family-specific variants, we focused on nonsynonymous variants that were not contained in dbSNP, of which there were 531. We then looked at variants where the two affected children and the mother had at least one minor allele, but the unaffected children and the father had at least one less minor allele compared to the mother and affected children. There were 14 variants that met these criteria, however, four of these variants were identified in the 1000 genomes project and were excluded leaving ten variants for further consideration (Table 4). In all cases, affected individuals were heterozygous and unaffected individuals were homozygous wild-type. Based on the functional prediction scores (two or more algorithms indicate a high probability of functionality (marked with an asterisk in Table 4), four variants in four different genes were deemed functionally interesting (*PDE4DIP*, *CBLB*, *KALRN*, *GALNTL6*). No mutation was predicted to be fuctional by all programs, and none by more than three of the five programs. The isoleucine to leucine change in *PDE4DIP* was predicted to be functional by Mutation Taster (high probability of a disease causing site) and LRT (evidence of codon constraint). The *CBLB* mutation of aspartic acid to alanine was predicted to be functional by PhyloP (highly conserved among 46 vertebrate species), Mutation Taster and LRT. The leucine to phenylalanine mutation in *KALRN* was supported by PhyloP and LRT, whereas the isoleucine to valine mutation in *GALNTL6* was supported by SIFT (high probability of a damaging change) and PhyloP. Three of these genes (*PDE4DIP*, *CBLB*, and *KALRN*) are of increased interest based on either their function or previous work in asthma. All three SNPs were re-genotyped using TaqMan and for each SNP the mother and both affected children were heterozygous, while the father and two unaffected children were homozygous wild-type (Table 5). In addition, we genotyped four SNPs that have been reported to be associated with asthma, rs7216389 in *ORMDL3*, rs11684634 in *PDE11A*, rs1544791 in *PDE4D* and rs2706347 in *RAD50* (Table 5). We did not observe the risk allele segregating with the affection status for any of these “common” asthma variants.

**Table 4:** Family-specific variants identified in mother and two affected children and variant annotations from ANNOVAR

				**Functional prediction program**				
**Gene**	**Amino acid change**^**1**^	**Chr**^**2**^	**Pos**^**3**^	**SIFT**^**4**^	**PolyPhen2**^**5**^	**PhyloP**^**6**^	**Mutation taster**^**7**^	**LRT**^**8**^
PDE4DIP	I303L	1	144930802	0.47	0.45	0.75	1.00^*^	0.95^*^
FCRL6	R123Q	1	159778799	0.14	0.28	0.02	0.01	0.00
AIM2	N194D	1	159035936	0.00	0.99^*^	0.16	0.08	0.69
ZBTB37	M396V	1	173854936	0.00	--	--	--	--
IER5	E103K	1	181058345	0.07	0.07	0.94	0.02	0.91
CBLB	D454A	3	105438937	0.37	0.78	0.99^*^	0.99^*^	0.99^*^
CCDC80	S95L	3	112358469	0.13	0.03	6.2E-4	0.04	6.0E-6
KALRN	L1644F	3	124281690	0.00	0.83	0.99^*^	0.72	0.99^*^
GALNTL6	I160V	4	173269765	1.00^*^	0.00	0.99^*^	0.00	0.84
COMMD5	R38Q	8	146076611	0.47	0.03	0.10	0.06	0.94

^1^Amino acid changes are represented as the wild type amino acid, position of the amino acid in the polypeptide chain followed by the amino acid resulting from the mutation. All amino acids are represented by the single letter amino acid code.

^2^Chr = Chromosome.

^3^Pos = chromosome position.

^4^SIFT, prediction of a change being damaging (>0.95) or tolerated. Values >0.95 are marked with an asterisk. For all five functional prediction programs, missing values (−−) were not provided by ANNOVAR.

^5^PolyPhen2, prediction of a change being damaging (>0.85), possibly damaging (0.15-0.85) or benign (<0.15). Values >0.85 are marked with an asterisk.

^6^Phylop, prediction of a conserved (>0.95) or non-conserved site (<0.95) sites across species. Values >0.95 are marked with an asterisk.

^7^Mutation Taster, prediction of a disease-causing variant (1 – p-value). Values >0.95 are marked with an asterisk.

^8^LRT, likelihood ratio test for codon constraint. Values >0.95 are marked with an asterisk.

**Table 5:** Genotypes at exome sequence candidate SNPs and four additional asthma associated SNPs

**Family member**	**Asthma phenotype**	**CBLB (G)**^**1**^	**KALRN (T)**^**1**^	**PDE4DIP (C)**^**1**^	**ORMDL3 (T)**^**1**^	**RAD50 (T)**^**1**^	**PDE11A (C)**^**1**^	**PDE4D (T)**^**1**^
Mother	Affected	GT	CT	AC	TT	GT	AA	CC
Father	Unaffected	TT	CC	AA	CT	GT	AC	CT
Child 1	Affected	GT	CT	AC	CT	TT	AC	CT
Child 2	Affected	GT	CT	AC	CT	TT	AC	CC
Child 3	Unaffected	TT	CC	AA	CT	TT	AC	CT
Child 4	Unaffected	TT	CC	AA	CT	GG	AA	CT

^1^Minor or risk allele is in parentheses.

To determine if the asthmatic children carried more rare variants in asthma candidate genes than non-asthmatic children, we queried the 251 asthma candidate genes from our previous study [23] to determine the number of variants each child in this family had across this set of genes. This was done for all variants regardless of function or frequency, only variants classified as novel (not observed in dbSNP), and only novel and nonsynonymous variants. While one of the affected children had the most number of rare alleles across candidate genes (n=427), one of the unaffected children had the second highest number of rare alleles (n=422). However, when we limited this to either novel variants or novel and nonynonymous variants, the affected children had more rare alleles than unaffected children in both categories (Table 6), but this difference did not reach statistical significance for either novel (p=0.112) or novel and nonsynonymous (p=0.097) when compared to the total number of variants passing QC among the affected and unaffected children. Despite the lack of statistical significance the direction of the association indicated that the number of rare variants increased risk among both the novel (OR=1.964; 95%CI: 0.84-4.58) and novel and nonsynonymous (OR=2.357; 95%CI: 0.83-6.69).

**Table 6:** Number of minor alleles across 251 asthma candidate genes

**Family member**	**Asthma phenotype**	**All variants**^**1**^	**Novel variants**	**Novel and nonsynonymous variants**
Child 1	Affected	415	8	6
Child 2	Affected	427	8	6
Child 3	Unaffected	396	3	1
Child 4	Unaffected	422	5	4

^1^Varants passing QC filters.

One advantage that we had sequencing an entire nuclear family was the ability to identify *de novo* variants, variants identified in the children not observed in either parent. We identified 279 *de novo* variants that were present in one or more of the children, but not in the parents that passed our initial QC. Overall these variants had low coverage with an average of 13.5X and drastically higher than the < 1 event per subject observed in recent studies [24,25]. When we imposed strict quality control criteria (see Methods) to distinguish true positive *de novo* events from false positive, this was reduced to three *de novo* variants in three subjects. However, it is reasonable to assume that *de novo* events are likely not to have been previously ovbserved. Therefore, we restricted this to only variants not in dbSNP, leaving two *de novo* variants (Table 7). These two variants had high overall coverage with an average of 212X across the six family members. These two variants were both nonsynonymous mutations, the first in the gene *DST* in a non-asthmatic child and the second in the gene *MEF2A* in an asthmatic child.

**Table 7:** ***De novo *variants**

					**Mother**		**Father**		**Child 1**		**Child 2**		**Child 3**		**Child 4**	
**Function**	**Gene**	**Exonic function**	**Chr**^**2**^	**Position**	**Gen**^**3**^	**Reads**^**4**^	**Gen**^**3**^	**Reads**^**4**^	**Gen**^**3**^	**Reads**^**4**^	**Gen**^**3**^	**Reads**^**4**^	**Gen**^**3**^	**Reads**^**4**^	**Gen**^**3**^	**Reads**^**4**^
exonic	MEF2A	nonsyn^1^	15	100252908	0/0	155,0	0/0	187,0	0/1	73,65	0/0	223,0	0/0	76,0	0/0	196,3
exonic	DST	nonsyn^1^	6	56485113	0/0	221,0	0/0	333,1	0/0	248,0	0/0	326,0	0/0	145,0	0/1	168,122

^1^Nonsynonymous.

^2^Chromosome.

^3^Genotype (0/0 = homozygous reference, 0/1 = heterozygous for the *de novo* variant, 1/1 = homozygous *de novo*).

^4^Number of reads covering the variant site with the first number being the number of reads containing the reference allele and the second the number of reads containing the *de novo* variant allele.

## Discussion

We recruited one family in which the mother and two children reported a doctor’s diagnosis of asthma, but the father and two additional children had no doctor’s diagnosis of asthma. After subjecting each family member’s genome to whole exome sequencing, we identified ten novel, nonsynonymous variants that segregated perfectly with asthma. Three of these variants had high probability to result in deleterious protein coding changes by two or more functional prediction algorithms and were also plausible asthma candidate genes based on their function or previous asthma association studies.

*PDE4DIP*, also known as myomegalin, is a golgi associated protein that is found to interact with a member of the phosphodiesterase superfamily of proteins, *PDE4D*, in the sarcomeric structure of skeletal muscle [26]. It has been shown to regulate cardiac contractility through its activity by phosphorylating Cardiac Myosin Binding Protein-C (*cMyBPC*) and cardiac troponin (*cTNI*) [27]. While no studies have shown expression of *PDE4DIP* in lung tissue, interest in this gene potentially playing a role in asthma susceptibility stems from the fact that it interacts with *PDE4D*. A GWAS identified an association between childhood asthma and variants in *PDE4D* and was replicated in several populations [3].

*CBLB* codes for the protein E3 ubiquitin ligase Cbl-b a member of the Cbl family of proteins. Of particular interest is its reported role in the regulation of the T cell response through promotion of the clearance of the T-cell receptor from the cell surface [28]. It has previously been reported that a region of chromosome 7 containing the T-cell receptor gamma (*TCRγ*) gene is associated with asthma [29,30].

*KALRN* codes for the protein kalirin. Reduced levels of kalirin have been associated with increased inducible nitric oxide synthetase (*iNOS*) activity [31] and promoter polymorphisms in the *iNOS* gene have been associated with allergic asthma severity [32]. Evidence for an involvement of *KALRN* is further supported by the finding of a nominal association with a single-nucleotide polymorphism in *KALRN* in our previously published GWAS of childhood asthma [16].

The focus of GWAS has been on identifying common risk variants associated with disease. However, recent large-scale sequencing studies suggest that the majority of variation present in the genome is rare, and estimates of frequency of novel variants are as high as 82% [33,34]. Given that the largest proportion of variants in the genome may be novel, we chose to focus our attention on variants not present in variant databases. It can be argued that there may be rare, but not novel, variants segregating with asthma in this family, but at least the large consortium-based GWAS for asthma was likely sufficiently powered to identify associations with even these rare variants [2]. This allowed us to focus on variants that might only be identified through the use of a family-based design.

As expected, common risk variants in four asthma candidate genes did not segregate with asthma in this family. It appears as if one of the unaffected children (child 4) had fewer risk alleles (n=2) than the other children in this family (each had four or more), but it is not possible to conclude if this can account for the phenotypic differences. However, we did observe that the affected children had more rare alleles within a wider array of asthma candidate genes compared to the unaffected children. While this data is inconclusive, there appears to be a trend of more rare variants in asthma candidate genes among case children than control children suggesting that a combination of rare variants across multiple genes may be contributing to asthma susceptibility. This is a hypothesis that will need to be tested in a larger dataset.

One advantage of this family-based design is that we had the ability to identify *de novo* mutation events that could be potentially contributing to asthma susceptibility in the affected children or protecting against asthma in the unaffected children. We identified two novel variants in two genes, *DST* and *MEF2A*, that had high coverage and could potentially be risk, *MEF2A*, or protective, *DST*, asthma variants. Both of these variants were nonsynonymous variants, but neither were in genes previously associated with asthma or had an obvious function relationship with asthma. *MEF2A* codes for a DNA-binding transcription factor and is primarily found in proliferating smooth muscle cells in humans [35]. Mutations in *MEF2A* have been associated with with coronary artery disease (CAD) [36,37], however, further evidence suggests that this is not a common cause of CAD among whites [38]. *DST* codes for the protein, dystonin, a member of the plankin family of proteins, whose members differentially express splice variants involved in the cytoskeletal needs of various specialized cells [39]. A homozygous single base pair deletion in *DST* was identified as the causal mutation in a family segregating a lethal form of heredity sensory autonomic neuropathy (HSAN) [40].

While many more families will be needed to demonstrate the involvement of any of these genes in asthma, this is the first report of a comprehensive examination of exonic variants within a family segregating asthma and one of the first to describe this for a common, non-Mendelian disease. We chose to focus here on family-specific variants, but asthma development is likely influenced by both family-specific variants in addition to more common asthma susceptibility variants. Future studies will need to focus on identifying family-specific variants across families that are all either within the same gene or within genes in the same genetic pathway. Finally, these family-specific variants will need to be analyzed within the context of common asthma susceptibility variants, the majority of which are non-exonic, to yield a more comprehensive view of genetic contributors to asthma across a range of effect sizes. Whole-genome sequencing will enable such future work.

## Conclusions

This is the first report applying exome sequencing to identify asthma susceptibility variants. Despite having sequenced only one family segregating asthma, we identified several potentially functional variants in three interesting asthma candidate genes, *PDE4DIP*, *CBLB* and *KALRN*. This will provide the basis for future work in which more families will be sequenced to identify variants across families that cluster within genes.

## Competing interests

The authors do not have any conflicts of interest, financial or otherwise.

## Author’s contributions

ATD, KMW and MBB conceived and designed the study. KBE, KH and KS recruited subjects. NP performed all laboratory work. ATD performed all data analysis. ATD and MBB interpreted the results. ATD drafted the manuscript. All authors revised the manuscript for important intellectual content, read and approved the final manuscript.

## Pre-publication history

The pre-publication history for this paper can be accessed here:

http://www.biomedcentral.com/1471-2350/13/95/prepub
